# Lightweight ZnO/Carbonated Cotton Fiber Nanocomposites for Electromagnetic Interference Applications: Preparation and Properties

**DOI:** 10.3390/polym16010116

**Published:** 2023-12-29

**Authors:** Muhammad Waseem, Yuxiang Xie, Kesong Yu, Xiling Zhou, Yingchun Cai, Xiaoli Zhang, Baochen Liu, Jingbo Chen

**Affiliations:** 1 School of Materials Science and Engineering, National Engineering Research Center for Advanced Polymer Processing Technology, Zhengzhou University, Zhengzhou 450001, China; waseemshoukat296@gmail.com (M.W.);; 2 School of Water Conservancy and Transportation, Zhengzhou University, Zhengzhou 450001, China; 3 Guangdong Provincial Key Laboratory of Technique and Equipment for Macromolecular Advanced Manufacturing, South China University of Technology, Guangzhou 510610, China

**Keywords:** cotton fiber, zinc oxide, carbonation, electromagnetic interference shielding, composites

## Abstract

Electromagnetic wave pollution has become a significant harm posed to human health and precision instruments. To shelter such instruments from electromagnetic radiation, high-frequency electromagnetic interference (EMI) shielding materials are extremely desirable. The focus of this research is lightweight, high-absorption EMI shielding composites. Simple aqueous dispersion and drying procedures were used to prepare cotton fiber (CF)-based sheets combined with various zinc oxide (ZnO) contents. These composites were carbonated in a high-temperature furnace at 800 °C for two hours. The obtained CF/ZnO samples have densities of 1.02–1.08 g/cm^3^. The EMI shielding effectiveness of CF-30% ZnO, CF-50% ZnO, and CF-70% ZnO reached 32.06, 38.08, and 34.69 dB, respectively, to which more than 80% of absorption is attributed. The synergetic effects of carbon networks and surface structures are responsible for the high EMI shielding performance; various reflections inside the interconnected networks may also help in improving their EMI shielding performance.

## 1. Introduction

With the prompt development of radar detection technology, wireless telecommunications, and related electronic equipment, the electromagnetic field unavoidably generates pollution, which has turned out to be one of the most severe problems, matching noise, water, and air pollution [1,2,3,4,5]. To solve the induced electromagnetic interference (EMI) problems, different applications of microwave-absorbing resources (MARs) have garnered considerable attention in the commercial and military fields because of their ability to absorb and shield against electromagnetic radiation [6,7,8,9,10]. In recent years, various types of EMI shielding materials, such as electrically lightweight conductive polymer composites (CPCs), MXenes, electrically conductive filled textiles, and paper-shaped materials in harsh environments, have been reported and compared to their metal-type counterparts [11,12,13,14,15,16].

In comparison to their predecessors, newly applied EMI shielding composites are more flexible, lighter, and more capable of tuning their conductivity through subsequent processes [17,18,19,20]. Due to their simplicity of preparation, light weight, low cost, flexibility, high electromagnetic shielding effectiveness, and corrosion resistance, the ideal EM wave absorption materials must generally possess strong absorption properties. Because of this, traditional ferrite composites, Fe_3_O_4_, CuO, and ZnO-based composites have unique advantages over traditional metal materials [21,22,23]. These advantages include higher effective complex permittivity, better corrosion resistance, a higher servicing temperature, and relatively low density, which may be related to their synergetic EMI shielding effects and microwave absorption properties [21,24,25,26]. Thus, zinc oxide (ZnO) is a promising option to satisfy the abovementioned specifications. The scientific community has been interested in ZnO-based nanocomposite (NC) materials, which have a wide range of uses in LEDs, transparent conductors, gas sensing, UV shielding, and other areas, with relatively advanced dielectric properties [2,27,28,29,30]. As one of the most popular inorganic materials used in medicine, chemical engineering, the plastics industry, etc., ZnO nanoparticles are suitable substitutes with ideal properties, including a high specific surface area, high electron mobility, high-temperature resistance, and chemical stability. ZnO-based electroconductive composites have promising features due to their outstanding benefits, which include low cost, facile preparation, and excellent service condition adaptability [24,25,26,31,32].

To obtain the dielectric loss effect in EMI shielding composites, metal fibers and carbons (carbon black, carbon fiber, carbon nanotube, and graphene) are usually used to construct electric conductive circuits inside the materials [11,33,34,35]. Porous cellulose fibers are abundantly available, renewable natural resources that are usually used in the production of paper and in the pulp-making industry. After carbonization, they represent cheap and abundant carbon resources that can be applied in the EMI shielding field. Recently, it was shown that nanocomposites including ZnO, such as ZnO-coated iron nano-capsules, Fe_3_O_4_/shell magnetic/ZnO core nanoparticles, and ZnO nanowire polyester composites, can be used in microwave absorption. ZnO has a unique three-dimensional (3D) structure that has drawn increasing attention because of its superior mechanical, chemical, and thermal stability [36]. Generally speaking, developing ZnO/cellular-based conductive paper is the primary response to improve the absorption effectiveness of such EMI shielding material. It is possible to produce cellular papers with high electric conductivity through a high-temperature sintering process, as referenced in CPC reports of frequently used CNTs and GNPs [11,14,37,38,39,40]. For example, Wang et al. recently researched a rGO-WPU/cotton fabric hybrid composite, which exhibits flexible, durable, and thermally conducting properties with robust electromagnetic interference shielding performance [41]. Filter sheets have reportedly been used as models for electromagnetic interference shielding; however, they used carbon nanofiber as the electrically conductive media.

Due to cotton fiber’s low cost, non-toxicity to the skin, and simplicity of manufacturing, we have prioritized it over ZnO nanostructures like CF/ZnO as a high-efficiency material [42,43,44]. In this paper, we put forth a novel technique to protect against electromagnetic wave pollution in the manufacture of cotton fiber/ZnO-based highly conductive, lightweight, flexible, and high-temperature composites. A facile process that is analogous to the typical wet paper-making action was implemented, using carbonated cotton fiber as a special electrically conductive source. The properties of the resultant EMI shielding materials were also enhanced.

## 2. Experimental

### 2.1. Materials

The National Pulp and Paper Research Institute of China distributed cotton fiber (white color) with an average thickness of 15.0–20.0 μm and an average length of 2.0–3.0 mm, which was initially planted in Hebei province of China. Following the removal of lignin and colloids and three stages of bleaching, the obtained pulp cotton fibers were produced with a majority of the cellulose and semi-cellulose remaining [42,45]. ZnO with a purity of 99.5% was purchased from Aladdin Chemistry Company of China (Shanghai, China).

### 2.2. Preparation of Cotton Fiber/ZnO-Based EMI Shielding Materials

The need for specialized applications in high-temperature environments, such as electromagnetic compatibility in challenging environments, has drawn particular attention to high-temperature electromagnetic (EM)-absorbing and EMI shielding materials. Preparing high-performance electromagnetic shielding materials, which are primarily used to protect against electromagnetic wave emission sources, is the most efficient way to eradicate the dangers of electromagnetic (EM) waves. The addition of ZnO to the carbonated cotton fiber, as previously noted, may achieve high electric conductivity, which is a key component of the multifunctional mechanisms of high-performance EMI shielding composites.

**Figure 1:**
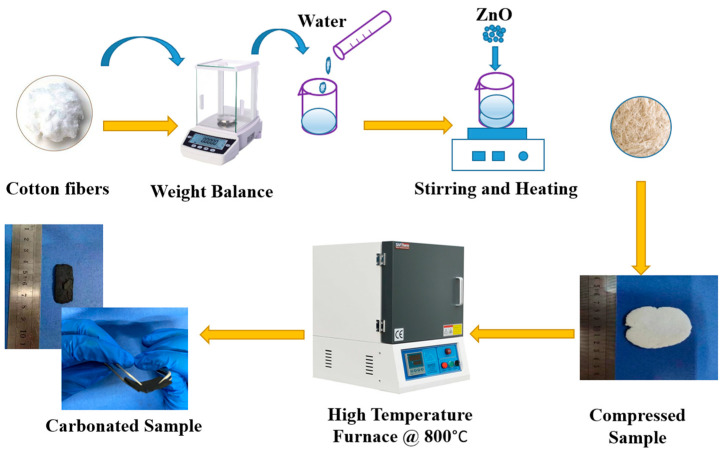
Schematic diagram of the sample preparation.

Another important element that should be properly taken into account while producing EMI shielding composites is the surface feature [46]. The prepared cotton fiber samples with various concentrations of sintered ZnO (CF-0% ZnO, CF-10% ZnO, CF-30% ZnO, CF-50% ZnO, and CF-70% ZnO) were then examined and compared in terms of their ability to protect against EMI. The detailed fabrication process for the cotton-fiber-based EMI shielding nanocomposites is schematically illustrated in Figure 1.

To produce cellulose papers with high electrical conductivity, 1.5 g of weighted cotton fibers was first added to 80 mL of deionized water to produce a suspension of cotton fibers (CFs), to which varying amounts of ZnO (ZnO contents of 0, 10, 30, 50, and 70% and ZnO and cotton fiber weights of 0 g, 0.15 g, 0.45 g, 0.75 g, and 1.5 g, 1.35 g, 1.05 g, 0.75 g, and 0.45 g) weighed using an electrical balance, were added while undergoing heating by ultrasonic stirring. The resulting CF/ZnO suspensions were then heated to 100 °C to evaporate the water, and porous cakes were obtained using a variety of samples. After several heating operations in a vacuum oven (DZF-6020A, Lichen Bangxi Instrument and Technology Company, Shanghai, China) at 100 °C for 4 h each, the CF/ZnO-based papers were pressed (XLB-D300x300/0.25 MN, Qingdao Xinben Science and Technology Co. Ltd., Qingdao, China) into a circular shape with an average diameter of 60.0 mm and a thickness of about 1.00 mm under a pressure of 10 MPa at room temperature (25 °C) and a humidity of 30% before a constant measurement. To prepare carbonated samples, the pressed samples were loaded into a high-temperature furnace (BR-17M-5, produced by Zhengzhou Brother Furnace Company, Zhengzhou, China) at 800 °C for 2 h, after which samples were collected intermittently. The weight–content ratios of the various zinc oxide components in the samples are marked. For instance, the sample encryption of CF-0% ZnO, CF 10% ZnO, CF-30% ZnO, CF-50% ZnO, and CF-70% ZnO refers to five different samples of cotton fiber composite treated with ZnO, as shown in Table 1.

The ZnO contents of the individual samples were 0, 10, 30, 50, and 70 wt%, which representing their unique ideal properties and characteristics. As shown in Table 1, additional samples are also prepared in accordance with their concentrations before being compressed collectively using the same laboratory press.

### 2.3. Characterizations

A field emission scanning electron microscope (SEM) (The EM-30 Plus COXEM Company, Daejeon, Republic of Korea) was used to examine the morphologies of the generated ZnO-containing conductive cotton-fiber-based sheets, along with the various samples of nanocomposite materials. The Fourier transform infrared spectroscopy (FTIR) spectra (Nicolet 6700, Thermo Scientific, Waltham, MA, USA) were recorded with a resolution of 4 cm^−1^ using a KBr tablet. A Tektronix DMM-4050 (Beaverton, OR, USA) was used to measure the electrical conductivities of the cellulose conductive sheets. Two face-to-face waveguide-to-coaxial adaptors in the frequency range of 18.0–28.0 GHz were used in conjunction with an Agilent N5234A vector network analyzer system (VNAS, Agilent Scientific Instruments, Gainesville, GA, USA) to examine the effectiveness of the EMI shielding. Before the measurements, the VNAS scattering limitations were calibrated. The samples under evaluation were cut into a rectangular shape with dimensions of 4.3 mm in width and 10.6 mm in length to fix the waveguide holders. The coefficients of transmittance (T), absorbance (A), and reflectance (R) often characterize the EMI SE of the material. The sum of electromagnetic radiation multiple reflections (SE_M_), absorption (SE_A_), and reflection (SE_R_), defined as the SE total, is the transmitted power quantity measurement in decibels (dB). S_21_ and S_11_ were used to derive EMI shielding factors SE absorption, SE reflection, and SE total (SE_A_, SE_R_, and SE_T_, respectively) as Formulas (1)–(6) [47].

T = |S_21_|^2^ (1)

R = |S_11_|^2^ (2)

A = 1 − T − R(3)

(4) $${\mathrm{SE}}_{A}=-10\;\log\;[T/1-R]$$

SE_R_ = −10 log (1–R) (5)

SE_T_ = SE_A_ + SE_R_ + SE_M_ (6)

## 3. Results and Discussions

### 3.1. Morphologies of CF/ZnO Nanocomposites before and after Carbonization

**Figure 2:**
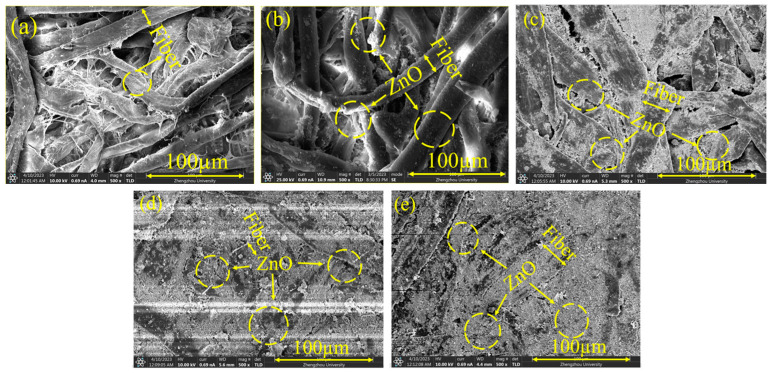
SEM images at the same magnification: (**a**) CF-0% ZnO, (**b**) CF-10% ZnO, (**c**) CF-30% ZnO, (**d**) CF-50% ZnO, and (**e**) CF-70% ZnO before carbonization.

Based on the simple preparation technique for the cotton fiber distribution and sintering process with or without the introduction of ZnO nanoparticles, cotton fibers were used to construct the connected network inside the comparatively flexible sheets. Figure 2 represents the surface and cross-sectional morphological characteristics of the produced sheets. The surfaces of the CF-0% ZnO, CF-10% ZnO, CF-30% ZnO, CF-50% ZnO, and CF-70% ZnO composites are shown in Figure 2a–e before carbonization. As indicated by the arrows, curly cotton fibers overlap with each other due to their considerable flexibility and their micrometer diameter. After drying, the fibers were in tight contact. With the addition of ZnO nanoparticles, the abundant hydrogen bonds of the cotton fiber helped to adsorb the ZnO on the surface [48,49]. Furthermore, when the ZnO content was increased to 30% or more, gaps or holes between the cotton fibers were filled by these nanoparticles, as can be clearly seen in the SEM images, influencing their EMI shielding performance after further carbonization.

**Figure 3:**
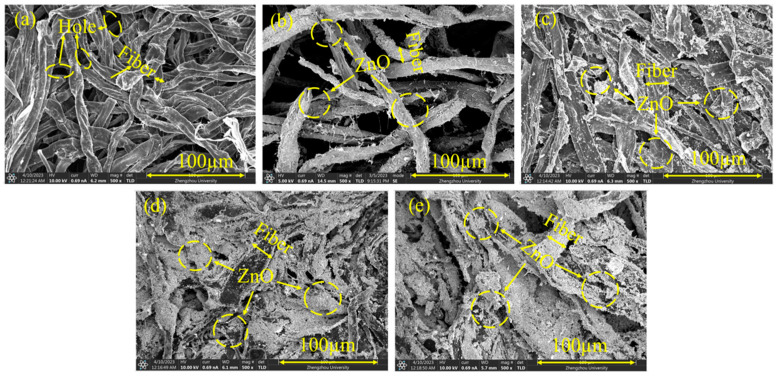
SEM images at the same magnification: (**a**) CF-0% ZnO, (**b**) CF-10% ZnO, (**c**) CF-30% ZnO, (**d**) CF-50% ZnO, and (**e**) CF-70% ZnO after carbonization.

The surface of CFs became stiff after carbonization compared with the original fibers, and different contents of ZnO were deposited on CFs to achieve high-performance EMI shielding with high electrical conductivity. Figure 3a–e show SEM images of the morphologies of CF-0% ZnO, CF-10% ZnO, CF-30% ZnO, CF-50% ZnO, and CF-70% ZnO conductive papers after carbonization. The ZnO particles compactly stuck to the cotton fiber surface, indicating good interfacial interaction between ZnO particles and cotton fibers. As mentioned above, CFs include several hydroxyl groups, making it simple for ZnO to be absorbed on the surfaces of small cellulose cotton fibers. The relatively high radiance brightness of the ZnO overlay sheets shown in in Figure 3 demonstrates the conductive qualities of the carbonated samples, which, assisted by the fine ZnO distribution structure, aided in the production of electrical channels or conductive circuits. In contrast to the unique three-dimensional ideal structures of ZnO, comparatively flat cotton fiber surfaces with irregular fiber circulation can be identified in Figure 3.

Consequently, different dark-colored samples of CF-0% ZnO, CF-10% ZnO, CF-30% ZnO, CF-50% ZnO, and CF-70% ZnO after carbonization of networks with densities of 1.02–1.08 g/cm^3^ were produced with significant weight loss and volume contraction. In comparison to CF-0% ZnO, the carbonated composites displayed relative flexibility, as presented previously in Figure 1. In the higher-temperature carbonization furnace, the CF/ZnO networks formed with cotton fiber displayed comparable distinctive but more tightly packed structures, exhibiting porous three-dimensional (3D) conductive frameworks composed of multiple conductive fibers with decreasing diameter. The SEM images after carbonization also clearly show that CF/ZnO is a very promising lightweight electromagnetic absorber due to its strong electronic conductivity and large specific surface area. The CF/ZnO nanocomposites with different concentrations of ZnO and cotton fiber (CF) are deep black in color after carbonation.

### 3.2. FTIR Analysis

**Figure 4:**
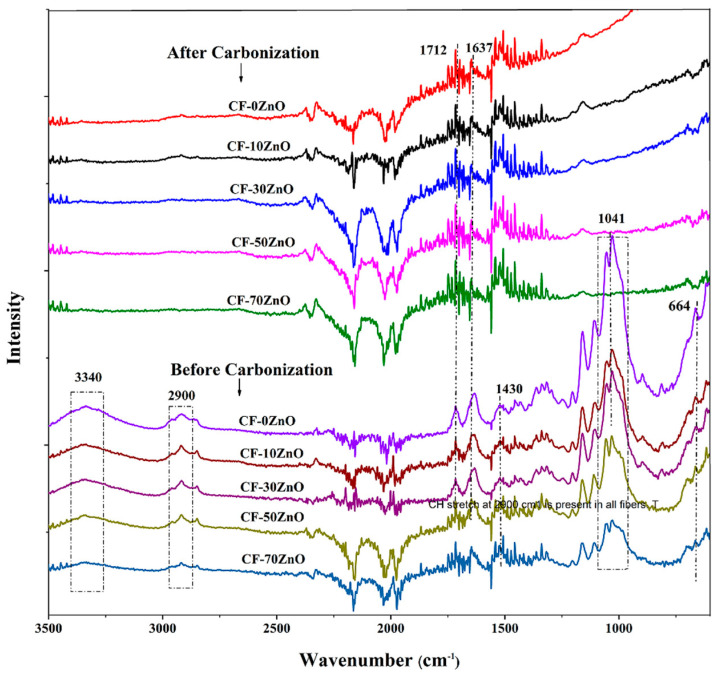
FTIR spectra of CF/ZnO samples before and after carbonization.

Since FTIR analysis can be highly beneficial in establishing a plausible picture of typical bonds and their interactions in composites, FTIR testing is also effective, quick, non-destructive, and able to provide comprehensive information about the sample’s molecular structure. To examine CF/ZnO functional groups and the changing of hydroxyl groups in cotton fibers both before and after carbonization treatment, the FTIR spectra of all samples (CF-0% ZnO, CF-10% ZnO, CF-30% ZnO, CF-50% ZnO, and CF-70% ZnO) are shown in Figure 4.

The stretching or bending vibrations of hydroxyl groups in cotton fibers before carbonization were associated with the peaks in the spectra of cotton fiber/ZnO that clearly emerged at 664, 1040, 1430, and 3340 cm^−1^ [50,51]. Cellulose and hemicellulose are the main contents of the cotton fibers treated by alkali. Before the carbonization treatment performed in this study, the bending vibration of O-H with high cotton fiber loading presented a strong peak at 664 cm^−1^, as shown in Figure 4, while 1430 cm^−1^ is the bending vibration of O-H and the peaks at 1040 and 3340 cm^−1^ correspond to the CH_2_-OH of cellulose. Moreover, the peak at 2900 cm^−1^ is attributed to the existence of the C-H stretching vibration [52]. After carbonization, most of these peaks disappeared, corresponding to the elimination of the O-H groups. Furthermore, the peaks at 1712 and 1637 cm^−1^ are attributed to the presentation of C=O and C=C groups of hemicellulose, which are associated with stretching vibrations [51,52]. After carbonization, broadened peaks appeared around 1565 cm^−1^, indicating the existence of a carbon skeleton. A mild van der Waals interaction between cotton fiber and ZnO in the composite is suggested by the spectra’s increased intensity and small shift in characteristic frequencies after carbonization of the cotton fibers.

### 3.3. Performance of Electric Conductive Papers with Carbonated Cellulose Fibers

**Figure 5:**
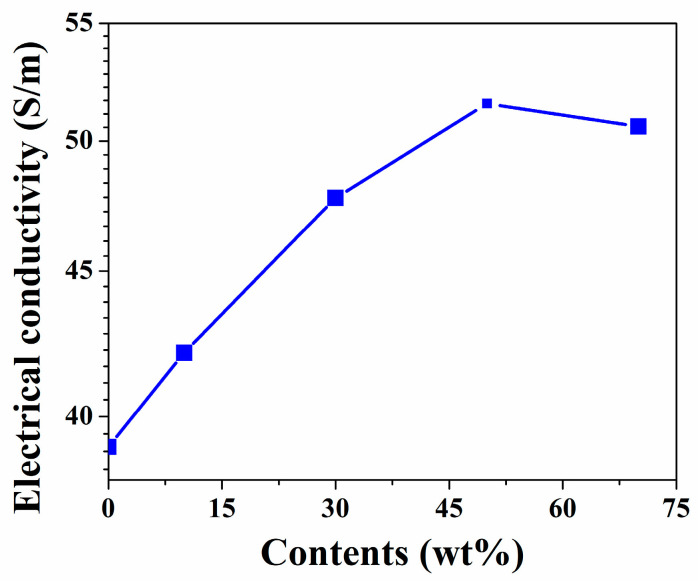
Electric conductivities of CF-0% ZnO, CF-10% ZnO, CF-30% ZnO, CF-50% ZnO, and CF-70% ZnO.

Figure 5 shows the different electric conductivities of conductive papers (CF-0% ZnO, CF-10% ZnO, CF-30% ZnO, CF-50% ZnO, and CF-70% ZnO) after carbonization. Carbonated cotton fibers are responsible for the electrical conductivity performance of the composites. When incorporated with semiconductive ZnO, it is abundantly obvious that the electrical conductivity of CF/ZnO-based papers slightly increased, owing to the increasing contents of ZnO (0, 10, 30, 50, and 70%). The maximum conductivity value of CF-50% ZnO was 53 S/m, but the electrical conductivity started to decline to below 50 S/m at 70% ZnO content because less cotton fiber and excessive amounts of zinc oxide were utilized in this case.

**Figure 6:**
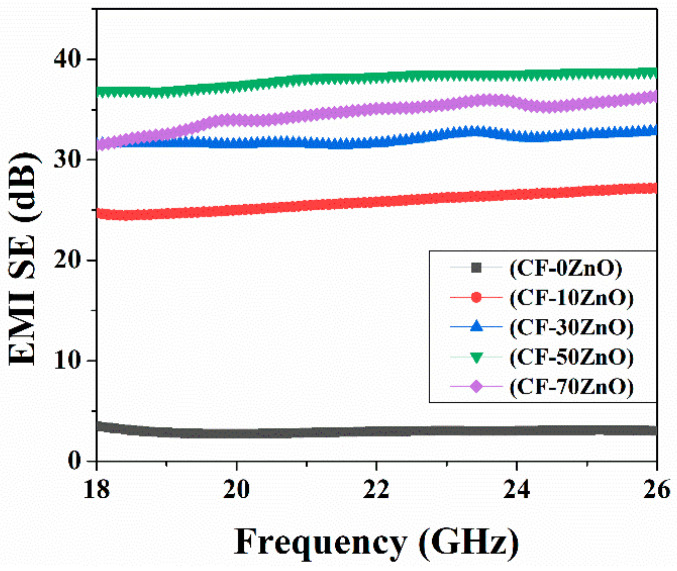
EMI shielding effectiveness of CF-0% ZnO, CF-10% ZnO, CF-30% ZnO, CF-50% ZnO, and CF-70% ZnO.

The tested SE values for the series of conductive papers (CF-0% ZnO, CF-10% ZnO, CF-30% ZnO, CF-50% ZnO, and CF-70% ZnO) with a thicknesses of 3.6 to 4.0 mm (three layers) are shown in Figure 6. These values are relatively high (over 20.0 dB), making them perfect for commercial EMI shielding applications. With the increase in ZnO loading, the number of semiconductive absorption sites increased, and the huge holes between the carbonized fibers were filled, resulting in an increase in EMI shielding effectiveness.

The CF-70% ZnO, CF-50% ZnO, CF-30% ZnO, and CF-10% ZnO samples were found to have SE_T_ values of 34.08, 38.08, 32.06, and 25.88 dB, respectively, in the range of 18–26 GHz; these values are clearly higher than that of CF-0% ZnO, only 2.96 dB, which means that ZnO is semiconductive, although carbonated cotton fibers made the greatest contribution in terms of electrical conductivity in the present study, resulting in the superior EMI shielding performances of CF-ZnO composites. Relatively large holes or concaves existed in the carbonated pure cotton fiber sheets, so electromagnetic waves could pass through these cells easily, which resulted in relatively ineffective EMI shielding performance.

EMI efficiencies were enhanced by more than 20.0 dB in this research utilizing varied concentrations of ZnO. With increasing ZnO concentrations, such defects could be amended; conductive channels or circuits were constructed to induce dielectric loss, which was found to be the key factor in improving EMI shielding performance in this study. The sample with 50% added ZnO corresponded to the highest SE_T_ value of 38.08 dB, indicating a balance between the components and shielding performance. Too much ZnO addition (70%) corresponds to weakening or damage to electric circuits and, thus, a decrease in EMI shielding efficiency.

It should be emphasized that absorption dominates all other types of shielding. A comparison of the EMI shielding performance of the composites (CF-0% ZnO, CF-10% ZnO, CF-30% ZnO, CF-50% ZnO, and CF- 70% ZnO) is shown in Table 2. The SE_A_, SE_R_, and SE_T_ calculations of these samples show that absorption mechanisms are still predominant in the CF/ZnO-based EMI shielding nanocomposites, also showing the absolute absorption dominance of CF/ZnO (more than 80%). In comparison to CF-70% ZnO, CF-50% ZnO, CF-30% ZnO, and CF-10% ZnO, with a distribution gradient of CF-0% ZnO in the conductive network, it was possible to rearrange the layers and cause multiple reflections. Due to the presence of large interfaces and surfaces between carbonated fibers and ZnO particles, multiple reflections could be induced by increasing the transmission path of incident electromagnetic waves (EMWs) to achieve high shielding efficiency through the filler–matrix interface and the layer interface so that the EMWs presented multiple reflections and transmissions until they were absorbed and dissipated according to the type of heat [53]. However, when the distance between reflecting surfaces or interfaces is comparable or longer than the skin depth, these multiple reflections can be ignored or ascribed to the absorption component [53,54]. The EMI shielding performances of the electrically conductive sheets determined in this section are comparable to those recommended in the literature for practical applications [55]. The highest shielding effectiveness (SE) value among the tested layered conductive papers (CF-70% ZnO, CF-50% ZnO, CF-30% ZnO, and CF-10% ZnO) is higher than that required for commercial applications [56,57].

### 3.4. EMI Shielding Mechanism and Performance of the CF/ZnO Composites

Due to their dense structure, conductive cellulose papers with a high concentration of ZnO composites can be employed to achieve optimal EMI shielding performance, as demonstrated in the current study. To further increase the performance of EMI shielding composites, different concentrations of ZnO were exploited to fill gaps between cotton fibers at high temperatures (800 °C) in five conductive sheets (CF-0% ZnO, CF-10% ZnO, CF-30% ZnO, CF-50% ZnO, and CF-70% ZnO). ZnO was found to decrease the direct penetration of EM incident waves through concaves and holes. When we constructed carbonated samples, the circles and concaves shown in Figure 2 and Figure 3 were filled with this non-toxic material to improve their EMI shielding performance. The electromagnetic interference (SE) of the cotton-fiber-based nanocomposite conductive sheet was also noticeably improved, assisting in the construction of conductive channels or circuits. Mass surfaces between carbonized fibers and ZnO nanoparticles may also have inner reflection effects, which can also increase their EMI shielding performance.

The stiff fiber formations could be easily differentiated because the cotton fibers were firmly linked in the ZnO basement, which could absorb energy throughout the fracture process, as indicated by the arrows in the Figure 2 and Figure 3. The composites initially had a somewhat rough, cracked cross section. It is interesting to note that throughout the whole test frequency range, the SE_T_ values of CF-70% ZnO, CF-50% ZnO, CF-30% ZnO, and CF-10% ZnO were significantly greater than that of CF-0% ZnO. Additionally, the nanoparticle distribution was enhanced, influencing the dielectric loss and the efficient surface effect. As a result, the average SE_T_ values of the CF-70% ZnO, CF-50% ZnO, CF-30% ZnO, and CF-10% ZnO samples were substantially greater than that of CF 0% ZnO, which leads to the conclusion that the synergetic consequence of the multiple reflections and dielectric loss on the interlayered surfaces resulted in the increase in the shielding effectiveness value to 38.08 dB of CF-50% ZnO compared to coated equivalents in the same frequency range [58].

These consequences also prove our estimations; the calculated SE_T_ value of CF-0% ZnO is considerably lower than those of CF-10% ZnO, CF-30% ZnO, CF-50% ZnO, and CF-70% ZnO. It is obviously that nanoparticles are essential to achieve strong EMI shielding performance with CF/ZnO at various concentrations. The components of the EMI efficiency of composites are shown in Table 2. As expected, SE_A_ is the dominant shielding mechanism, which is advantageous in terms of preventing potential secondary pollution, which is mostly induced by reflection waves.

The multifunctional effects of special line-point-network structures (CF-10% ZnO, CF-30% ZnO, CF-50% ZnO, and CF-70% ZnO) that may cause multiple reflections owing to the various bulky surfaces and layers of randomly disposed cellulose cotton fibers can be attributed to these results. The tested composites (CF-0% ZnO, CF-10% ZnO, CF-30% ZnO, CF-50% ZnO, and CF-70% ZnO) are low-cost, flexible, and lightweight, making them viable materials for future EM radiation protection applications. Using the process outlined here, we were able to produce a lightweighted conductive nanocomposite that can achieve excellent performance in EMI shielding by reconstructing numerous interfaces in the composites.

**Figure 7:**
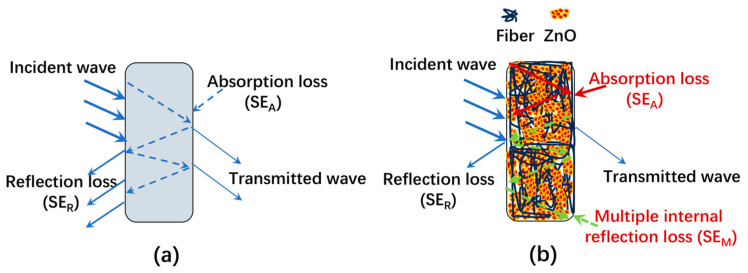
Schematic illustration of the EMI shielding mechanism: (**a**) typical EMI shielding mechanism of traditional materials; (**b**) multiple EMI shielding mechanism proposed in this study.

In traditional EMI shielding material, a portion of the electromagnetic wave that travels through the shielding material is reflected on the outer surface (SE_R_), but the remaining portion of the wave penetrates through the shielding material and continues to be transmitted (Figure 7a). In this study, at two shielding material interfaces, the EM wave was repeatedly transmitted and reflected while being attenuated by the EMI shielding material during the process of transmission. As a result, the absorption loss of the shielding material, reflection loss on the shielding material’s surface, and numerous internal reflection losses inside the shielding material contribute to the shielding mechanism of EMI shielding materials against EM waves (Figure 7b). The three-dimensional mass surfaces and structures of ZnO can assist in extending the transition range of electrons in both the cross-sectional and plane directions of ZnO-coated cotton fibers.

In other words, through “tunneling” effects, mass surfaces provide opportunities for various scatterings and internal reflections both in and around the cellulose cotton fibers and the inter layered surfaces of ZnO [59]. High conductivity or a high dielectric constant is required for a shielding barrier. The radiation is reflected and absorbed numerous times by the substance to provide shielding. Due to imperfect impedance matching, some electromagnetic waves that strike a material’s surface are reflected back, and some of the waves that pierce the material are lost due to dielectric loss, while the remaining waves are transmitted. We can therefore conclude that the strong dielectric characteristics, increased scattering interfaces, and numerous reflections of the connected network of CF/ZnO substantially improved microwave absorption performance. A schematic representation of the electromagnetic interference shielding mechanism based on the discussion above is presented in Figure 7. The overall EMI SE was considerably improved and dominated by an absorption mechanism, as illustrated in Table 3. After carbonization, our produced CF-ZnO composites have the obviously advantages of cheap, facile, and green processing; absorption dominance; and a relatively high level of EMI shielding effectiveness.

## 4. Conclusions

In summary, in this study, we investigated EMI shielding effectiveness and electrical conductivity in relation to the SE of CF/ZnO composites. The resulting cotton fiber demonstrated improved capacity for heat transmission, excellent electrical conductivity, and improved EMI shielding performance with decreasing diameter under an elevated carbonization temperature as a result of the formation of a conductive interconnected network. The majority of the manufactured CF/ZnO samples achieved an EMI shielding performance of about 80%. In comparison to CF-0% ZnO, which only achieved 2.96 dB in terms of EMI performance, the sandwich structures of the CF-70% ZnO, CF-50% ZnO, CF-30% ZnO, and CF-10% ZnO nanocomposites produced achieved maximum EMI performances of 34.69, 38.08, 32.06, and 25.88 dB, respectively, in the frequency range of 18–26 GHz. The synergistic effects of impedance matching, scattering, multiple reflections, and dielectric loss led to outstanding EMI shielding efficiency. Consequently, dark samples of carbonated networks were obtained with considerable volume shrinkage and weight loss; their densities were also calculated to be between 1.02–1.08 g/cm^3^. The experimental results and characteristics of CF-0% ZnO, CF-10% ZnO, CF-30% ZnO, CF-50% ZnO, and CF-70% ZnO show that ZnO and carbonated fibers are the most influential synergistic materials. By achieving a more dependable, constant network and channel within conductive fillers, ZnO and cotton fibers (CFs) were found achieve synergy, resulting in improved electrical conductivity. In comparison to samples filled with CF-0% ZnO nanoparticles, the EMI shielding performance of the ZnO-coated composites was clearly improved by the filling of micropores with cotton fibers. The experimental findings and percolation characteristics reported in this study demonstrate that ZnO-coated nanocomposite fibers are suitable materials for specialized EM shielding applications. The formed fibers are thin, porous, light, and flexible, and they are made with cost-effective components. The approach for developing the EM shielding fibers described in this research allows for further characterization and optimization of fibers for applications in electronics and communications, as well as military, aerospace, and environmental protection applications, among others.

## Figures and Tables

**Table 1:** Concentration of ZnO according the related mass ratio.

Name	Zinc Oxide (g)	Cotton (g)	Sample (g)
CF-0% ZnO	0	1.5	1.5
CF-10% ZnO	0.15	1.35	1.5
CF-30% ZnO	0.45	1.05	1.5
CF-50% ZnO	0.75	0.75	1.5
CF-70% ZnO	1.05	0.45	1.5

**Table 2:** Shielding effectiveness values of CF/ZnO (SE_A_, SE_R_, and SE_T_).

ZnO Weight (%)	Concentration	SE_A_	SE_R_	SE_T_
0	CF-0% ZnO	2.93	0.03	2.96
10	CF-10% ZnO	20.12	5.75	25.88
30	CF-30% ZnO	26.32	5.73	32.06
50	CF-50% ZnO	28.94	5.74	34.69
70	CF-70% ZnO	33.55	4.5	38.08

**Table 3:** EMI shielding performances of carbon (MXene)-filled composites.

Materials	Carbon Contents (wt.%)	Thickness (mm)	EMI SE (dB)	SE_A_ (dB)	Frequency Range (GHz)	Reference
rGO-ZnO	rGO 40	1	38	16.71	8–12.4	[24]
MoS2-rGO/ZnO	rGO 17.5	1.5	32	24	8.2–12.4	[25]
ZnO-MXene Ti_3_C_2_T_x_	Ti_3_C_2_T_x_ 70	4	26.3	10	10–18	[36]
cellulose nanofiber/polyaniline	Fiber 50	0.28	25.2	20.1	8–12.4	[49]
rGH/epoxy	rGO 1.2	2	38	6	8–12.4	[19]
Polyaniline-WCNH/PVDF	WCNH 1	2	29.7	17.5	14.5–20	[60]
CF-50% ZnO	Fiber 50	3.6	38.08	28.94	18–26.5	This work

## Data Availability

The authors confirm that the data supporting the findings of this study are available within the article.
